# Supplementary material for: Addressing the mental health needs of healthcare professionals in Africa: a scoping review of workplace interventions
Source: Glob Ment Health (Camb). 2025 Feb 26;12:e31. doi: 10.1017/gmh.2025.19 (PMC11894417; doi:10.1017/gmh.2025.19)
Supplement: Harri et al. supplementary material [file S2054425125000196sup001.docx]

Appendix 1: Search Strategy

| **Database** | **Search Strategy** |
| --- | --- |
| **MEDLINE**  **Ovid MEDLINE(R) ALL** 1946 to August 03, 2023 | 1. (mental health or psychiatric or psychological or wellbeing or well-being or wellness or distress or depression or anxiety or stress or burnout or "burn out" or resilien* or cope or coping or post-traumatic stress or posttraumatic stress or PTSD or work-life or compassion fatigue).ti,ab,kf.  2. exp Mental Health/ or Resilience, Psychological/  3. exp Stress, Psychological/  4. exp Occupational Stress/  5. exp Mental Fatigue/  6. Stress Disorders, Post-Traumatic/  7. Depression/  8. exp Anxiety/  9. or/1-8  10. exp Health Personnel/ or (acupuncturist* or allergist* or anatomist* or an?esthesiologist* or anesthetist* or audiologist* or cardiologist* or chiropractor* or clinician* or dentist* or dermatologist* or diabetologist* or dietician* or doctor* or doula or doulas or endocrinologist* or gastroenterologist* or general practitioner* or geriatrician* or gynecologist* or h?ematologist* or ((health or healthcare or hospital or medical) adj2 (worker* or workforce or personnel or practitioner* or provider* or professional* or employee* or staff* or navigator* or officer*)) or hospitalist* or immunologist* or intensivist* or internist* or medical resident* or midwife or midwives or neonatologist* or nephrologist* or neurologist* or neurosurgeon* or nurse or nurses or nutritionist* or obstetrician* or oncologist* or ophthalmologist* or optometrist* or osteopath or osteopaths or otolaryngologist* or pathologist* or p?ediatrician* or pharmacist* or pharmacologist* or phlebotomist* or physician* or podiatrist* or prosthetist* or psychologist* or psychiatrist* or pulmonologist* or radiographer* or radiologist* or radiotherapist* or rheumatologist* or surgeon* or therapist* or toxicologist* or urologist* or veterinarian*).mp.  11. ((mental health or health promot* or wellbeing or well being or wellness or occupational health) adj7 (job or jobs or workplace or worksite* or "work site*" or workforce or employee* or employer* or worker* or personnel or staff* or hospital* or clinic*1 or laborator* or pharmacy or pharmacies or dispensar* or "place of work" or "medic* cent*" or "medic* store*" or chemist*1 or "health* cent*" or "care cent*" or "health* facilit*")).mp.  12. exp Occupational Health Services/  13. employee assistance program*.mp.  14. or/11-13  15. exp Africa/ or (Africa* or Algeria or Angola or Benin or Botswana or "Burkina Faso" or Burundi or "Cabo Verde" or "Cape Verde" or Cameroon or "Central African Republic" or Chad or Comoros or Congo or "Cote d'Ivoire" or "Ivory Coast" or Djibouti or Egypt or "Equatorial Guinea" or Eritrea or Eswatini or Ethiopia or Gabon or Gambia or Ghana or Guinea or "Guinea-Bissau" or Kenya or Lesotho or Liberia or Libya or Madagascar or Malawi or Mali or Mauritania or Mauritius or Morocco or Mozambique or Namibia or Niger or Nigeria or Rwanda or "Sao Tome" or Principe or Senegal or Seychelles or "Sierra Leone" or Somalia or "South Africa" or Sudan or Swaziland or Tanzania or Togo or Tunisia or Uganda or Zambia or Zimbabwe).mp.  16. 9 and 10 and 14 and 15  17. limit 16 to english language |
| **Embase**  **Ovid Embase** 1974 to 2023 August 03 | 1. (mental health or psychiatric or psychological or wellbeing or well-being or wellness or distress or depression or anxiety or stress or burnout or "burn out" or resilien* or cope or coping or post-traumatic stress or posttraumatic stress or PTSD or work-life or compassion fatigue).ti,ab,kw.  2. exp mental health/ or psychological resilience/  3. exp mental stress/  4. exp job stress/  5. exp mental fatigue/  6. posttraumatic stress disorder/  7. depression/  8. exp anxiety/  9. or/1-8  10. exp health care personnel/ or (acupuncturist* or allergist* or anatomist* or an?esthesiologist* or anesthetist* or audiologist* or cardiologist* or chiropractor* or clinician* or dentist* or dermatologist* or diabetologist* or dietician* or doctor* or doula or doulas or endocrinologist* or gastroenterologist* or general practitioner* or geriatrician* or gynecologist* or h?ematologist* or ((health or healthcare or hospital or medical) adj2 (worker* or workforce or personnel or practitioner* or provider* or professional* or employee* or staff* or navigator* or officer*)) or hospitalist* or immunologist* or intensivist* or internist* or medical resident* or midwife or midwives or neonatologist* or nephrologist* or neurologist* or neurosurgeon* or nurse or nurses or nutritionist* or obstetrician* or oncologist* or ophthalmologist* or optometrist* or osteopath or osteopaths or otolaryngologist* or pathologist* or p?ediatrician* or pharmacist* or pharmacologist* or phlebotomist* or physician* or podiatrist* or prosthetist* or psychologist* or psychiatrist* or pulmonologist* or radiographer* or radiologist* or radiotherapist* or rheumatologist* or surgeon* or therapist* or toxicologist* or urologist* or veterinarian*).mp.  11. ((mental health or health promot* or wellbeing or well being or wellness or occupational health) adj7 (job or jobs or workplace or worksite* or "work site*" or workforce or employee* or employer* or worker* or personnel or staff* or hospital* or clinic*1 or laborator* or pharmacy or pharmacies or dispensar* or "place of work" or "medic* cent*" or "medic* store*" or chemist*1 or "health* cent*" or "care cent*" or "health* facilit*")).mp.  12. exp occupational health service/  13. employee assistance program*.mp.  14. or/11-13  15. exp Africa/ or (Africa* or Algeria or Angola or Benin or Botswana or "Burkina Faso" or Burundi or "Cabo Verde" or "Cape Verde" or Cameroon or "Central African Republic" or Chad or Comoros or Congo or "Cote d'Ivoire" or "Ivory Coast" or Djibouti or Egypt or "Equatorial Guinea" or Eritrea or Eswatini or Ethiopia or Gabon or Gambia or Ghana or Guinea or "Guinea-Bissau" or Kenya or Lesotho or Liberia or Libya or Madagascar or Malawi or Mali or Mauritania or Mauritius or Morocco or Mozambique or Namibia or Niger or Nigeria or Rwanda or "Sao Tome" or Principe or Senegal or Seychelles or "Sierra Leone" or Somalia or "South Africa" or Sudan or Swaziland or Tanzania or Togo or Tunisia or Uganda or Zambia or Zimbabwe).mp.  16. 9 and 10 and 14 and 15  17. limit 16 to english language |
| **PsycInfo**  **APA PsycInfo** 1806 to July Week 4 2023 | 1. (mental health or psychiatric or psychological or wellbeing or well-being or wellness or distress or depression or anxiety or stress or burnout or "burn out" or resilien* or cope or coping or post-traumatic stress or posttraumatic stress or PTSD or work-life or compassion fatigue).ti,ab.  2. exp Mental Health/  3. exp Psychological Stress/  4. exp Occupational Stress/  5. exp Compassion Fatigue/  6. Posttraumatic Stress Disorder/  7. "Depression (Emotion)"/  8. exp Anxiety/  9. or/1-8  10. exp Health Personnel/ or (acupuncturist* or allergist* or anatomist* or an?esthesiologist* or anesthetist* or audiologist* or cardiologist* or chiropractor* or clinician* or dentist* or dermatologist* or diabetologist* or dietician* or doctor* or doula or doulas or endocrinologist* or gastroenterologist* or general practitioner* or geriatrician* or gynecologist* or h?ematologist* or ((health or healthcare or hospital or medical) adj2 (worker* or workforce or personnel or practitioner* or provider* or professional* or employee* or staff* or navigator* or officer*)) or hospitalist* or immunologist* or intensivist* or internist* or medical resident* or midwife or midwives or neonatologist* or nephrologist* or neurologist* or neurosurgeon* or nurse or nurses or nutritionist* or obstetrician* or oncologist* or ophthalmologist* or optometrist* or osteopath or osteopaths or otolaryngologist* or pathologist* or p?ediatrician* or pharmacist* or pharmacologist* or phlebotomist* or physician* or podiatrist* or prosthetist* or psychologist* or psychiatrist* or pulmonologist* or radiographer* or radiologist* or radiotherapist* or rheumatologist* or surgeon* or therapist* or toxicologist* or urologist* or veterinarian*).mp.  11. ((mental health or health promot* or wellbeing or well being or wellness or occupational health) adj7 (job or jobs or workplace or worksite* or "work site*" or workforce or employee* or employer* or worker* or personnel or staff* or hospital* or clinic*1 or laborator* or pharmacy or pharmacies or dispensar* or "place of work" or "medic* cent*" or "medic* store*" or chemist*1 or "health* cent*" or "care cent*" or "health* facilit*")).mp.  12. employee assistance program*.mp.  13. or/11-12  14. (Africa* or Algeria or Angola or Benin or Botswana or "Burkina Faso" or Burundi or "Cabo Verde" or "Cape Verde" or Cameroon or "Central African Republic" or Chad or Comoros or Congo or "Cote d'Ivoire" or "Ivory Coast" or Djibouti or Egypt or "Equatorial Guinea" or Eritrea or Eswatini or Ethiopia or Gabon or Gambia or Ghana or Guinea or "Guinea-Bissau" or Kenya or Lesotho or Liberia or Libya or Madagascar or Malawi or Mali or Mauritania or Mauritius or Morocco or Mozambique or Namibia or Niger or Nigeria or Rwanda or "Sao Tome" or Principe or Senegal or Seychelles or "Sierra Leone" or Somalia or "South Africa" or Sudan or Swaziland or Tanzania or Togo or Tunisia or Uganda or Zambia or Zimbabwe).mp.  15. 9 and 10 and 13 and 14  16. limit 15 to english language |
| **Cochrane Library**  via Wiley | #1 ("mental health" or psychiatric or psychological or wellbeing or well-being or wellness or distress or depression or anxiety or stress or burnout or "burn out" or resilien* or cope or coping or "post-traumatic stress" or "posttraumatic stress" or PTSD or work-life or "compassion fatigue"):ti,ab,kw  #2 [mh "Mental Health"] or [mh ^"Resilience, Psychological"]  #3 [mh "Stress, Psychological"]  #4 [mh "Occupational Stress"]  #5 [mh "Mental Fatigue"]  #6 [mh "Stress Disorders, Post-Traumatic"]  #7 [mh ^Depression]  #8 [mh Anxiety]  #9 {OR #1-#8}  #10 [mh "Health Personnel"] or acupuncturist* or allergist* or anatomist* or an?esthesiologist* or anesthetist* or audiologist* or cardiologist* or chiropractor* or clinician* or dentist* or dermatologist* or diabetologist* or dietician* or doctor* or doula or doulas or endocrinologist* or gastroenterologist* or general practitioner* or geriatrician* or gynecologist* or h?ematologist* or ((health or healthcare or hospital or medical) NEAR/2 (worker* or workforce or personnel or practitioner* or provider* or professional* or employee* or staff* or navigator* or officer*)) or hospitalist* or immunologist* or intensivist* or internist* or medical resident* or midwife or midwives or neonatologist* or nephrologist* or neurologist* or neurosurgeon* or nurse or nurses or nutritionist* or obstetrician* or oncologist* or ophthalmologist* or optometrist* or osteopath or osteopaths or otolaryngologist* or pathologist* or p?ediatrician* or pharmacist* or pharmacologist* or phlebotomist* or physician* or podiatrist* or prosthetist* or psychologist* or psychiatrist* or pulmonologist* or radiographer* or radiologist* or radiotherapist* or rheumatologist* or surgeon* or therapist* or toxicologist* or urologist* or veterinarian*  #11 ((health NEXT promot*) or "occupational health") AND (job or jobs or workplace or worksite* or (work NEXT site*) or workforce or employee* or employer* or worker* or personnel or staff* or "place of work" or (medic* NEXT cent*) or (health* NEXT cent*) or (care NEXT cent*) or (health* NEXT facilit*) )  #12 [mh "Occupational Health Services"]  #13 employee assistance program*  #14 {OR #11-#13}  #15 (Africa* or Algeria or Angola or Benin or Botswana or "Burkina Faso" or Burundi or "Cabo Verde" or "Cape Verde" or Cameroon or "Central African Republic" or Chad or Comoros or Congo or "Cote d'Ivoire" or "Ivory Coast" or Djibouti or Egypt or "Equatorial Guinea" or Eritrea or Eswatini or Ethiopia or Gabon or Gambia or Ghana or Guinea or "Guinea-Bissau" or Kenya or Lesotho or Liberia or Libya or Madagascar or Malawi or Mali or Mauritania or Mauritius or Morocco or Mozambique or Namibia or Niger or Nigeria or Rwanda or "Sao Tome" or Principe or Senegal or Seychelles or "Sierra Leone" or Somalia or "South Africa" or Sudan or Swaziland or Tanzania or Togo or Tunisia or Uganda or Zambia or Zimbabwe):ti,ab  #16 #9 AND #10 AND #14 AND #15 |
| **CINAHL** | S1 "mental health" or psychiatric or psychological or wellbeing or well-being or wellness or distress or depression or anxiety or stress or burnout or "burn out" or resilien* or cope or coping or post-traumatic stress or posttraumatic stress or PTSD or work-life or "compassion fatigue"  S2 (MH "Mental Health")  S3 (MH "Stress, Psychological+")  S4 (MH "Stress, Occupational+")  S5 (MH "Mental Fatigue+")  S6 (MH "Stress Disorders, Post-Traumatic+")  S7 (MH "Depression+")  S8 (MH "Anxiety+")  S9 S1 OR S2 OR S3 OR S4 OR S5 OR S6 OR S7 OR S8  S10 (MH "Health Personnel+")  S11 acupuncturist* or allergist* or anatomist* or an#esthesiologist* or anesthetist* or audiologist* or cardiologist* or chiropractor* or clinician* or dentist* or dermatologist* or diabetologist* or dietician* or doctor* or doula or doulas or endocrinologist* or gastroenterologist* or general practitioner* or geriatrician* or gynecologist* or h#ematologist* or ((health or healthcare or hospital or medical) N2 (worker* or workforce or personnel or practitioner* or provider* or professional* or emplo ...  S12 S10 OR S11  S13 (("mental health" or "health promot*" or wellbeing or "well being" or wellness or occupational health) N7 (job or jobs or workplace or worksite* or "work site*" or workforce or employee* or employer* or worker* or personnel or staff* or hospital* or clinic or clinics or laborator* or pharmacy or pharmacies or dispensar* or "place of work" or "medic* cent*" or "medic* store*" or chemist* or chemists or "health* cent*" or "care cent*" or "health* facilit*"))  S14 (MH "Occupational Health Services+")  S15 "employee assistance program*"  S16 S13 OR S14 OR S15  S17 (MH "Africa+") or (Africa* or Algeria or Angola or Benin or Botswana or "Burkina Faso" or Burundi or "Cabo Verde" or "Cape Verde" or Cameroon or "Central African Republic" or Chad or Comoros or Congo or "Cote d'Ivoire" or "Ivory Coast" or Djibouti or Egypt or "Equatorial Guinea" or Eritrea or Eswatini or Ethiopia or Gabon or Gambia or Ghana or Guinea or "Guinea-Bissau" or Kenya or Lesotho or Liberia or Libya or Madagascar or Malawi or Mali or Mauritania or Mauritius or Morocco or Mozambique or Namibia or Niger or Nigeria or Rwanda or "Sao Tome" or Principe or Senegal or Seychelles or "Sierra Leone" or Somalia or "South Africa" or Sudan or Swaziland or Tanzania or Togo or Tunisia or Uganda or Zambia or Zimbabwe)  S18 S9 AND S12 AND S16 AND S17  Limiters: English language |
| **Scopus** | TITLE-ABS-KEY ( "mental health" OR psychiatric OR psychological OR wellbeing OR well-being OR wellness OR distress OR depression OR anxiety OR stress OR burnout OR "burn out" OR resilien* OR cope OR coping OR "post-traumatic stress" OR "posttraumatic stress" OR ptsd OR work-life OR "compassion fatigue" ) AND TITLE-ABS-KEY ( acupuncturist* OR allergist* OR anatomist* OR an?esthesiologist* OR anesthetist* OR audiologist* OR cardiologist* OR chiropractor* OR clinician* OR dentist* OR dermatologist* OR diabetologist* OR dietician* OR doctor* OR doula OR doulas OR endocrinologist* OR gastroenterologist* OR "general practitioner*" OR geriatrician* OR gynecologist* OR h?ematologist* OR ( ( health OR healthcare OR hospital OR medical ) W/2 ( worker* OR workforce OR personnel OR practitioner* OR provider* OR professional* OR employee* OR staff* OR navigator* OR officer* ) ) OR hospitalist* OR immunologist* OR intensivist* OR internist* OR "medical resident*" OR midwife OR midwives OR neonatologist* OR nephrologist* OR neurologist* OR neurosurgeon* OR nurse OR nurses OR nutritionist* OR obstetrician* OR oncologist* OR ophthalmologist* OR optometrist* OR osteopath OR osteopaths OR otolaryngologist* OR pathologist* OR p?ediatrician* OR pharmacist* OR pharmacologist* OR phlebotomist* OR physician* OR podiatrist* OR prosthetist* OR psychologist* OR psychiatrist* OR pulmonologist* OR radiographer* OR radiologist* OR radiotherapist* OR rheumatologist* OR surgeon* OR therapist* OR toxicologist* OR urologist* OR veterinarian* ) AND TITLE-ABS-KEY ( ( ( "mental health" OR "health promot*" OR wellbeing OR "well being" OR wellness OR "occupational health" ) W/7 ( job OR jobs OR workplace OR worksite* OR "work site*" OR workforce OR employee* OR employer* OR worker* OR personnel OR staff* OR hospital* OR clinic OR clinics OR laborator* OR pharmacy OR pharmacies OR dispensar* OR "place of work" OR "medic* cent*" OR "medic* store*" OR chemist OR chemists OR "health* cent*" OR "care cent*" OR "health* facilit*" ) ) OR "employee assistance program*" OR "occupational health service*" ) AND TITLE-ABS-KEY ( africa* OR algeria OR angola OR benin OR botswana OR "Burkina Faso" OR burundi OR "Cabo Verde" OR "Cape Verde" OR cameroon OR "Central African Republic" OR chad OR comoros OR congo OR "Cote d&apos;Ivoire" OR "Ivory Coast" OR djibouti OR egypt OR "Equatorial Guinea" OR eritrea OR eswatini OR ethiopia OR gabon OR gambia OR ghana OR guinea OR "Guinea-Bissau" OR kenya OR lesotho OR liberia OR libya OR madagascar OR malawi OR mali OR mauritania OR mauritius OR morocco OR mozambique OR namibia OR niger OR nigeria OR rwanda OR "Sao Tome" OR principe OR senegal OR seychelles OR "Sierra Leone" OR somalia OR "South Africa" OR sudan OR swaziland OR tanzania OR togo OR tunisia OR uganda OR zambia OR zimbabwe ) AND ( LIMIT-TO ( LANGUAGE , "English" ) ) |
| **Web of Science Core Collection** | TS= ( "mental health" OR psychiatric OR psychological OR wellbeing OR well-being OR wellness OR distress OR depression OR anxiety OR stress OR burnout OR "burn out" OR resilien* OR cope OR coping OR "post-traumatic stress" OR "posttraumatic stress" OR ptsd OR work-life OR "compassion fatigue" )  AND TS= ( acupuncturist* OR allergist* OR anatomist* OR an?esthesiologist* OR anesthetist* OR audiologist* OR cardiologist* OR chiropractor* OR clinician* OR dentist* OR dermatologist* OR diabetologist* OR dietician* OR doctor* OR doula OR doulas OR endocrinologist* OR gastroenterologist* OR "general practitioner*" OR geriatrician* OR gynecologist* OR h?ematologist* OR ( ( health OR healthcare OR hospital OR medical ) NEAR/2 ( worker* OR workforce OR personnel OR practitioner* OR provider* OR professional* OR employee* OR staff* OR navigator* OR officer* ) ) OR hospitalist* OR immunologist* OR intensivist* OR internist* OR "medical resident*" OR midwife OR midwives OR neonatologist* OR nephrologist* OR neurologist* OR neurosurgeon* OR nurse OR nurses OR nutritionist* OR obstetrician* OR oncologist* OR ophthalmologist* OR optometrist* OR osteopath OR osteopaths OR otolaryngologist* OR pathologist* OR p?ediatrician* OR pharmacist* OR pharmacologist* OR phlebotomist* OR physician* OR podiatrist* OR prosthetist* OR psychologist* OR psychiatrist* OR pulmonologist* OR radiographer* OR radiologist* OR radiotherapist* OR rheumatologist* OR surgeon* OR therapist* OR toxicologist* OR urologist* OR veterinarian* )  AND TS= ( ( ( "mental health" OR "health promot*" OR wellbeing OR "well being" OR wellness OR "occupational health" ) NEAR/7 ( job OR jobs OR workplace OR worksite* OR "work site*" OR workforce OR employee* OR employer* OR worker* OR personnel OR staff* OR hospital* OR clinic OR clinics OR laborator* OR pharmacy OR pharmacies OR dispensar* OR "place of work" OR "medic* cent*" OR "medic* store*" OR chemist OR chemists OR "health* cent*" OR "care cent*" OR "health* facilit*" ) ) OR "employee assistance program*" OR "occupational health service*" )  AND TS= ( africa* OR algeria OR angola OR benin OR botswana OR "Burkina Faso" OR burundi OR "Cabo Verde" OR "Cape Verde" OR cameroon OR "Central African Republic" OR chad OR comoros OR congo OR "Cote d&apos;Ivoire" OR "Ivory Coast" OR djibouti OR egypt OR "Equatorial Guinea" OR eritrea OR eswatini OR ethiopia OR gabon OR gambia OR ghana OR guinea OR "Guinea-Bissau" OR kenya OR lesotho OR liberia OR libya OR madagascar OR malawi OR mali OR mauritania OR mauritius OR morocco OR mozambique OR namibia OR niger OR nigeria OR rwanda OR "Sao Tome" OR principe OR senegal OR seychelles OR "Sierra Leone" OR somalia OR "South Africa" OR sudan OR swaziland OR tanzania OR togo OR tunisia OR uganda OR zambia OR zimbabwe )  Languages: English |
